# Swift SNPs: Evaluating Rapid DNA Extraction Methods for Scalable Genotyping in Aquaculture

**DOI:** 10.1007/s10126-025-10561-7

**Published:** 2026-01-09

**Authors:** Emily Rhodes, Dean Jerry, David B. Jones, Julie Goldsbury, Nga Vu

**Affiliations:** Centre for Sustainable Tropical Fisheries & Aquaculture, The College of Science and Engineering, James Cook University, Townsville, QLD 4811 Australia

**Keywords:** DNA extraction, High-throughput genotyping, Target capture sequencing, HotSHOT, SNP genotyping, Aquaculture genomics

## Abstract

Efficient DNA extraction is critical for high-throughput genotyping in aquaculture breeding programs, where cost, speed, and data quality must be balanced. This study compared five rapid extraction methods—HotSHOT, Chelex, Tween, wax-coated dipstick, and bare dipstick—against a conventional cetyltrimethylammonium bromide (CTAB) protocol for use in *Lates calcarifer* (barramundi) genotyping. DNA yield, purity, and integrity were measured by spectrophotometry, fluorometry, and Agilent TapeStation. Performance was tested in microsatellite genotyping, tyrosinase-related protein 1b (TYRP1b) gene sequencing, and single nucleotide polymorphism (SNP) genotyping on the Tecan Allegro Targeted Resequencing V2 platform. CTAB produced the highest-quality DNA (30.3 ± 17.4 ng/µL; A260/A280 = 1.91 ± 0.26; DIN = 8.78) with reliable results in all assays. Despite lower quality (1.07 ± 0.30 ng/µL; A260/A280 = 1.44 ± 0.18; DIN ≈ 1.6), HotSHOT performed similarly: SNP concordance with CTAB averaged 82.3%, microsatellite amplification reached 93.1% (vs. 99.3% for CTAB), and all HotSHOT samples yielded high-quality TYRP1b sequences. Chelex, Tween, and dipstick methods had lower success, likely due to inadequate yield and purity. HotSHOT was the most effective rapid alternative to CTAB, with significantly higher SNP concordance (82.3% vs. others; *p* < 0.005). These results show that method choice strongly influences target-capture genotyping success and support HotSHOT as a cost-effective, scalable option for aquaculture genomics.

## **Introduction**

High-throughput single nucleotide polymorphism (SNP) genotyping is now fundamental to aquaculture breeding programmes, enabling the selection of traits such as growth, disease resistance, and environmental tolerance. These applications rely not only on robust genotyping platforms, but also on efficient and scalable DNA extraction protocols—an area that remains a major logistical and technical bottleneck.

Conventional SNP genotyping approaches include fixed-content microarrays and reduced-representation sequencing. SNP arrays, such as those on the Axiom™ platform (Thermo Fisher Scientific), genotype predefined variants using hybridization probes designed for specific SNPs. These arrays offer high accuracy, reproducibility, and cost-efficiency for large-scale genotyping. However, they lack flexibility, as they require prior knowledge of variant positions and are limited to the specific SNPs included on the array (Thermo Fisher Scientific, 2023), reducing their utility in non-model species with limited genomic resources (Morin et al., 2004). Additionally, SNP arrays cannot identify novel or population-specific variants not already assayed on the arrays. This limitation is particularly important in aquaculture breeding, where discovering new SNPs can identify markers linked to important traits or reveal genetic diversity unique to breeding populations.

In contrast, genotyping-by-sequencing (GBS) methods, such as double-digest RAD sequencing (ddRAD), generate reduced-representation libraries by digesting genomic DNA with two restriction enzymes and sequencing the resulting fragments. This approach enables simultaneous SNP discovery and genotyping based on sequence variation. Researchers can select restriction enzymes to target specific recognition sites, which in turn influences the number and distribution of sequenced loci across the genome. However, GBS is sensitive to restriction site polymorphisms. If a site is mutated in some individuals, the associated region may not be sequenced, leading to inconsistent locus coverage and missing genotype data (Andrews et al., 2016; Gautier et al., 2013). Additional challenges include PCR amplification bias and variable fragment size distributions, which can affect the depth and uniformity of sequencing. While GBS is advantageous for *de novo* studies in non-model species, its variability and high missing data rates make it less suitable for routine aquaculture breeding programs, which require consistent, reproducible, and high-throughput genotyping across large populations.

An emerging genotyping method is targeted-capture GBS, which combines the specificity of microarrays with the discovery potential and flexibility of sequencing. One such platform is the Allegro Targeted Genotyping V2 kit (Tecan Inc.), which employs Single Primer Enrichment Technology (SPET) to selectively amplify genomic regions flanking known targeted SNPs. In this method, a single extension primer binds just upstream of a target SNP and is extended by DNA polymerase to incorporate adjacent genomic sequence (~ 80–120 bp downstream of the probe), generating sequenceable fragments that include the SNP site and surrounding base pair sequence. This targeted design enables tens of thousands of SNPs to be analyzed in a single reaction. SPET provides a scalable solution: it minimizes missing data, reduces sequencing waste by avoiding off-target reads, and ensures consistent coverage across predefined markers critical for parentage, pedigree verification, and genomic selection.

Tecan’s guidelines for the Allegro platform recommend using 10 to 100 ng of intact (non-degraded) double-stranded DNA for optimal results (Tecan, 2022). “Intact” refers to high-molecular-weight DNA that has not undergone significant fragmentation, which typically corresponds to fragment sizes greater than 10 kilobases (kb) as visualized on an agarose gel or electropherogram. High-quality DNA is further defined by an A260:A280 ratio of 1.8 or above and an A260:A230 ratio of 2.0 or above, indicating low levels of protein and organic contaminants. The influence of DNA integrity and purity on SPET performance is not well characterized in aquaculture systems. The hybridization of primers and following extension and ligation can be compromised by degraded DNA, which may lack sufficient flanking sequence for reliable binding. Additionally, the presence of inhibitors such as salts, proteins, polysaccharides, or detergents can interfere with enzymatic activity during library construction (Head et al. 2014). Unlike standard SNP arrays, which rely on short-probe hybridization and tolerate moderate DNA fragmentation, or GBS methods, which intentionally sequence fragmented DNA, SPET workflows require a balance between DNA fragment length and purity to ensure efficient probe extension and accurate target capture.

The Allegro platform has been successfully applied in a variety of applications. In plant studies, maize samples processed with Allegro generated an average of 25 million reads and whole genome alignment rates of 88–91%, while eggplant libraries yielded over 30,000 high-confidence SNPs (Scaglione et al. 2019). In animal applications, the platform has been used to genotype feral horses and Rocky Mountain bighorn sheep from stool samples, demonstrating its flexibility and robustness across sample types (Gavriliuc et al. 2022; Deakin and Coltman 2024). However, this technology has had limited use to date in aquaculture species. An assay previously developed at James Cook University routinely surveys 5,000 SNPs; however, this assay is based on extraction of high-quality DNA using magnetic beads and/or CTAB, both laborious and expensive extraction protocols.

Numerous rapid DNA extraction protocols have been proposed, but they are often tailored to specific sample types or downstream applications, and their suitability for targeted-capture SNP genotyping in aquaculture species remains largely untested. Several comparative studies in aquaculture and fisheries have highlighted this gap. Li et al. (2017) reviewed genotyping-by-sequencing (GBS) applications across aquaculture and emphasized that DNA extraction remains a critical bottleneck, particularly in non-model species with high mucus or lipid content. Robledo et al. (2020) introduced a RAD-seq-based capture method and noted that DNA quality significantly impacted probe capture efficiency, further underscoring the need for quality DNA in targeted sequencing workflows. While some studies have compared extraction methods, they often focus on yield and purity rather than direct compatibility with GBS or targeted genotyping. For example, Chowdhury et al. (2016) assessed five extraction methods in two freshwater fish species and found trade-offs between yield and purity depending on the buffer system used but did not evaluate performance in downstream sequencing. Schiebelhut et al. (2017) compared eight extraction methods across eight animal phyla, finding that CTAB consistently produced high-quality DNA, though method performance varied widely among taxa, highlighting the importance of context-specific optimization. Similarly, Rodriguez-Riveiro et al. (2022) compared five extraction methods across marine fish and cephalopod taxa, finding significant differences in DNA yield and purity and highlighting trade-offs between cost, automation, and PCR success. These studies demonstrate that while alternative extraction methods to CTAB exist, few have been systematically evaluated for compatibility with targeted platforms like SPET.

This is particularly relevant for barramundi (*Lates calcarifer*), a fast-growing, euryhaline species that thrives in both freshwater and marine environments, making it highly suitable for aquaculture. Barramundi farming is expanding rapidly across Southeast Asia, Australia, and more recently in the Middle East and the Americas, with global production exceeding 130,000 tonnes annually (FAO 2024). To support the development of improved commercial strains, selective breeding programmes require genotyping thousands of individuals per generation to estimate breeding values, track pedigrees, and monitor genetic diversity. However, the high cost and labor demands of DNA extraction and SNP genotyping remain key barriers, especially when dealing with tissue types like fins. Efficient, low-cost DNA extraction methods compatible with high-throughput genotyping platforms are therefore essential to scaling genomic selection efforts in this species.

By systematically testing rapid DNA extraction methods for compatibility with high-throughput SNP genotyping in a commercially important aquaculture species, this study addresses a critical gap in current practice. The primary aim is to identify low-cost, scalable extraction protocols that maintain sufficient DNA quality for reliable genotyping, thereby reducing technical barriers to genomic selection and improving the efficiency of breeding programs. In addition to SNP genotyping using the Allegro platform, this study also includes microsatellite genotyping and sequencing of the Tyrosinase-related protein 1b (TYRP1b) gene. These PCR-based analyses remain widely used in aquaculture for applications such as parentage assignment and trait mapping, and their inclusion provides a broader assessment of how DNA quality impacts different downstream workflows. Collectively, these results will inform the selection of practical, cost-effective DNA extraction methods suitable for routine implementation in aquaculture genetics.

The six extraction methods evaluated here were selected because they represent the most commonly used non-kit, low-cost, and rapid alternatives to CTAB that are feasible for hatchery-scale workflows. Commercial extraction kits provide high-quality DNA but are cost-prohibitive when thousands of samples must be processed per generation. In contrast, HotSHOT, Chelex, Tween, and dipstick formats cost < AUD $1 per extraction, require minimal equipment, and can be implemented in 96-well or field-based formats. Although these methods are widely used in other taxa, their compatibility with targeted genotyping-by-sequencing platforms such as Allegro has not been systematically tested in aquaculture species. Establishing the performance limits of these rapid protocols is therefore essential for designing scalable breeding pipelines.

## Materials and Methods

### Sample Collection and Preparation

Fin clips were collected from barramundi (*Lates calcarifer*) sourced from a commercial hatchery (Mainstream Aquaculture Group, Werribee, Australia). The same 16 fin clips were used for each DNA extraction protocol. Fin samples previously extracted using the CTAB method and yielding DNA concentrations > 60 ng/µL (measured using the Quantifluor™ dsDNA System, Promega) were selected. These samples had also been genotyped using a custom 70 k Axiom™ myDesign™ SNP array (ThermoFisher Scientific™), providing known genotype profiles for each individual (Jerry et al. 2022). New portions from the same fin clips were then used for all extraction protocols to enable direct comparisons of DNA yield and quality across methods. Fin clips were stored in 95% ethanol at − 20 °C between the original CTAB-based extractions and the rapid extraction experiments described in this study. The storage duration was approximately 12 months. Despite prolonged storage, DNA integrity remained high in CTAB extracts, indicating that storage conditions did not introduce substantial degradation. All methods therefore began with tissue of similar preservation history, enabling direct comparison among extraction protocols.

All tissue samples used in this study were obtained from archived fin clips collected non-lethally by hatchery staff at Mainstream Aquaculture Group under their routine health monitoring and breeding protocols. No live animals were handled or euthanized specifically for this study. As such, ethical approval was not required. All procedures complied with institutional and national guidelines for the care and use of animals.

### DNA Extraction Protocols

A total of six DNA extraction protocols were evaluated in this study, including traditional, rapid, and dipstick-based methods (Table 1)

a) HotSHOT

The HotSHOT protocol is a rapid alkaline lysis method that requires minimal reagents, no organic solvents, and is highly scalable for high-throughput applications. Although it often yields DNA of lower purity and its performance can vary depending on tissue type, it is appropriate for PCR-based workflows due to its simplicity and efficiency (Truett et al. 2000). In this study, DNA was extracted using the HotSHOT protocol as originally described. A small piece of tissue (< 2 mm in diameter) was placed in a PCR tube containing 75 µL of alkaline lysis buffer (25 mM NaOH, 0.2 mM disodium EDTA, pH 12), then incubated at 95 °C for 1 h on a heating block. After cooling to 4 °C, an equal volume (75 µL) of neutralization buffer (40 mM Tris-HCl, pH 5) was added. This mixture also served as the DNA storage solution, with a final elution volume of 140 µL. Extracted samples were stored at 2 °C until further use

b) Wax-Coated Dipstick

The dipstick protocol developed by Zou et al. (2018) and Mason & Botella (2020) is described as an ultra-low-cost, equipment-free method well-suited for high-throughput or field-based DNA extractions. In this study, two variations of the protocol were tested: one using filter paper dipsticks coated in wax, and the other using bare filter paper strips without wax. Wax-coated dipsticks limit fluid uptake to a defined region, which may reduce contamination and improve reproducibility, while bare dipsticks offer a larger surface area for DNA binding. Although these methods typically yield less DNA and have not been widely validated in fish tissue, their affordability, speed, and minimal infrastructure requirements present compelling advantages for routine screening in aquaculture breeding programs.

To implement the protocol, Whatman No. 1 qualitative filter paper (11 μm pore size; Cytiva, Marlborough, MA, USA) was cut into 50 × 50 mm squares and partially submerged in melted Paraplast^®^ wax (Sigma-Aldrich, St. Louis, MO, USA) to coat approximately 40 mm of each strip. Once cooled, the waxed paper was trimmed into 2 mm × 44 mm strips, leaving a 2 × 4 mm unwaxed region at one end to act as the nucleic acid binding site. The reagent chemistry was adapted from Hammouda et al. (2019), originally applied to zebrafish and medaka embryos. Fin clips (~ 1 mg tissue) were incubated overnight at 55 °C in 800 µL of lysis buffer (0.4 M Tris-HCl pH 8.0, 5 mM EDTA, 0.15 M NaCl, 0.1% SDS) with 20 µL of Proteinase K (Astral Scientific). Wash buffer (Milli-Q water) and elution buffer (TE) were prepared in aliquots of 800 µL and 40 µL per sample, respectively. To extract DNA, each dipstick was immersed in the lysate and gently agitated for ~ 5 s, followed by five dips in the wash buffer with gentle wiping against the tube wall. The dipstick was then dipped into the elution buffer ~ 15 times, wiped again, and discarded. Eluted DNA was stored at 4 °C for downstream analysis.

c) Bare Dipstick

A variation of the dipstick method described by Mason and Botella (2020) and Zou et al. (2018) was implemented using uncoated strips of filter paper in place of wax-coated dipsticks. Whatman No. 1 filter paper was cut into 50 × 50 mm squares and then into strips measuring 2 mm in width and 44 mm in length, with no wax coating, allowing the entire surface area to serve as a nucleic acid binding site.

The same lysis buffer, incubation conditions, and reagent setup described for the wax-coated dipstick method were used. Prior to DNA binding, the dipsticks were folded back and forth approximately 15 times to form an accordion shape, maximizing surface area contact with the solution. Using tweezers, the folded dipsticks were lowered into the lysate and gently agitated for ~ 5 s. The dipsticks were then transferred to the wash buffer, gently compressed with tweezers for ~ 5 s, and wiped along the inner wall of the tube to remove excess liquid. Finally, the dipsticks were placed into the elution buffer and left in solution. Elution tubes were stored at 2 °C until further use.

d) CTAB

Genomic DNA was extracted from barramundi (*Lates calcarifer*) caudal fin tissue using a modified cetyltrimethylammonium bromide (CTAB) protocol originally described by Adamkewicz and Harasewych (1996), as adapted by Smith-Keune (2013). This method employs a CTAB lysis buffer and chloroform: isoamyl alcohol (24:1) extraction, followed by isopropanol precipitation. Approximately 100 mg of tissue was placed in a sterile 1.5 mL microcentrifuge tube containing 700 µL of preheated CTAB buffer (2% CTAB [Amresco, 0833-500G], 1.4 M NaCl, 100 mM Tris-HCl, pH 8.0, 20 mM EDTA) and 10 µL of Proteinase K (20 mg/mL; Astral Scientific). Samples were incubated at 65 °C for at least 1 h with occasional vortexing to facilitate tissue lysis and protein digestion. Lysates were purified via two sequential extractions with 700 µL of chloroform: isoamyl alcohol (24:1), centrifuged at 16,000 × g for 10 min at room temperature. The final aqueous phase was transferred to a new tube, and DNA was precipitated by adding 600 µL of cold isopropanol. Samples were incubated at − 20 °C for 1 h and centrifuged at 16,000 × g for 30 min. DNA pellets were washed with 1 mL of 70% ethanol, centrifuged again for 10 min at 4 °C, air-dried in a fume hood, and resuspended in 50–100 µL of 1× TE buffer (10 mM Tris-HCl, 0.1 mM EDTA, pH 8.0).

e) Tween

The Tween protocol, adapted from work in mollusks and fish embryos (Taris et al. 2005), balances simplicity and moderate DNA yield by using non-ionic detergents. However, its performance can vary depending on tissue type, limiting its reliability in some contexts. In this study, a modified Tween-20 extraction method was used, based on the original formulation by Taris et al. (2005). Fresh lysis buffer was prepared using 670 mM Tris-HCl (pH 8.0), 166 mM ammonium sulfate, 0.2% Tween-20 (Astral Scientific, BIO0777-500ML), and 0.2% IGEPAL CA-630 (Sigma-Aldrich, I3021-50ML). Proteinase K (20 mg/mL; Astral Scientific) was added at a ratio of 10 µL per 100 µL of buffer immediately before use. Tissue samples (~ 1 mm³) were incubated in 100 µL of this buffer with 5 µL of Proteinase K at 55 °C for 3–4 h to allow digestion, followed by heat inactivation at 95 °C for 10–20 min. Samples were stored at 2 °C and briefly centrifuged prior to use to pellet undigested debris.

f) Chelex

The Chelex protocol, widely used in forensic and field-based applications, is valued for its speed and low cost. It relies on a chelating resin to remove PCR inhibitors but may leave behind DNA-damaging metal ions and is not recommended for long-term DNA storage or applications requiring high molecular weight DNA (Walsh et al. 1991). In this study, a modified Chelex^®^ protocol was used, based on the original method by Walsh et al. (1991) and adapted by D.L. Rowe. A 5% Chelex solution was prepared fresh by dissolving 2.5 g of Chelex-100 resin (Sigma-Aldrich, Cat. No. 95577) in 50 mL of Milli-Q water. After thorough mixing to prevent settling, 500 µL of the suspension was dispensed into sterile 1.5 mL microcentrifuge tubes. A small tissue sample (~ 1 mm³) was added to each tube, followed by 2 µL of Proteinase K (20 mg/mL; Astral Scientific). The samples were briefly vortexed and incubated at 55 °C for approximately 2 h to allow digestion. Tubes were then vortexed again and heated at 95 °C for 8 min to inactivate Proteinase K and denature proteins. Following centrifugation at 10,000 × g for 5 min, the DNA-containing supernatant was carefully transferred to a new tube, avoiding disruption of the Chelex resin. Extracts were stored at 2 °C until further use.

**Table 1:** Comparison of six DNA extraction methods evaluated in this study, highlighting key differences in chemical reagents, equipment requirements, processing time, and cost. Total time and bench time assume a typical batch size of 24–48 samples

Protocol Name	Main Reagents	Equipment	Total time (hours)	Bench time (hours)	Cost per sample (AUD)
CTAB	Cetyltrimethylammonium bromide, chloroform: isoamyl, isopropanol	Centrifuge, fume hood	* 4–8	2–4	$5–6
HotSHOT	NaOH, Tris-HCl	Heat block	1–2	< 1	$0.30
Chelex	5% Chelex-100 resin, Proteinase K	Centrifuge, heat block	2–3	< 1	$1.50
Wax-coated Dipstick	Tris-HCl, EDTA, NaCl, SDS, Proteinase K	Heat block	* 4–5	1	$0.65
Bare dipstick	Tris-HCl, EDTA, NaCl, SDS, Proteinase K	Heat Block	* 4–5	1	$0.60
Tween	Tween 20, Tris-HCl, Proteinase K	Heat block, centrifuge	4–5	< 1	$0.30

* Total time increases if optional overnight step is included

### Quality Assessment

The quality of DNA from each extraction was evaluated based on visual observation, purity, concentration, and fragment length distribution. The purity of extracted DNA was assessed using a NanoDrop™ One spectrophotometer (Thermo Fisher Scientific, Waltham, MA, USA). DNA concentration (ng/µL) and absorbance ratios at 260/280 nm and 260/230 nm were measured using 2 µL of each sample. The A260/A280 ratio was used to assess protein contamination, with values between 1.8 and 2.0 typically indicating high-purity DNA. The A260/A230 ratio was used to evaluate contamination from salts, carbohydrates, and organic solvents; values above 2.0 were considered optimal.

DNA concentrations were measured using the Quantifluor^®^ dsDNA System (Promega Corporation, Madison, WI, USA) on a Zephyr^®^ automated liquid handling workstation (PerkinElmer, Waltham, MA, USA) in a 96-well plate format. Quantification was based on fluorescence emitted by the Quantifluor reagent upon binding specifically to double-stranded DNA. The fluorescence mode was selected over the “EnSpire” calculation setting, as it provided more accurate quantification for low-concentration samples. Each sample was measured in triplicate, and the mean concentration (ng/µL) was calculated. A standard curve was generated from a series of DNA standards with known concentrations included on each plate to ensure quantification accuracy. Accuracy was verified by comparing the measured values of the standards to their known concentrations.

The integrity and fragment size distribution of extracted DNA were assessed using the Agilent TapeStation (Agilent Technologies, USA). DNA samples (1 µL) were analyzed with the Genomic DNA ScreenTape assay following the manufacturer’s protocol. Samples were loaded into the TapeStation system, and electrophoretic traces were generated to determine the DNA Integrity Number (DIN). The DIN values, ranging from 1 (highly degraded) to 10 (highly intact), were used to compare DNA quality across extraction methods. The presence of high-molecular-weight bands and degradation patterns were also visually assessed in electropherograms.

### PCR-Based Analyses

a) Microsatellites

The efficacy of DNA extraction methods was assessed via microsatellite genotyping, which involved fluorescently labeled PCR amplification and fragment analysis. Microsatellites, or short tandem repeats (STRs), are highly polymorphic DNA regions consisting of repeating units of 2–6 base pairs. They are widely used in aquaculture for applications such as parentage analysis, genetic diversity assessment, and population structure studies—particularly in breeding programs for species like *L. calcarifer* (Yue et al. 2002; Loughnan et al., 2013; Loughnan et al. 2019). Fragment sizes typically range from 100 to 400 base pairs, making microsatellites suitable for analysis even when DNA is partially degraded. As a PCR-based technique, microsatellite genotyping is relatively tolerant of poor-quality DNA and provides a robust means of evaluating the suitability of DNA extraction methods for downstream molecular applications.

DNA samples were used without prior dilution. Nine microsatellite loci previously developed for *L. calcarifer* (Lca08, Lca20, Lca21, Lca58, Lca64, Lca69, Lca70, Lca74, and Lca98) were amplified using primers from Noble et al. (2014). Expected fragment sizes ranged from approximately 150–450 bp.

PCR reactions were prepared in a total volume of 9 µL per well, containing 5 µL of MyTaq™ HS Mix (Bioline, Meridian Bioscience, USA), 1 µL of a 10× primer mix, and 3 µL of molecular-grade water. An additional 1 µL of DNA template was added per reaction. Amplification was performed on a SimpliAmp™ thermal cycler (Thermo Fisher Scientific) under the following cycling conditions: initial denaturation at 95 °C for 5 min; 10 cycles of 95 °C for 30 s, 57 °C for 90 s, and 72 °C for 30 s; followed by 20 cycles with the annealing temperature reduced to 55 °C. A final extension was carried out at 60 °C for 3 min, followed by a 12 °C hold.

Post-PCR purification was conducted using Sephadex™ G-50 columns (GE Healthcare) to remove unincorporated primers and excess reagents. Purified products were sent to the Australian Genome Research Facility (AGRF) for capillary electrophoresis. Alleles were scored using GeneMapper^®^ Software v5 (Applied Biosystems), with manual review and adjustment for low-quality or ambiguous calls.

b) Gene Sequencing

Fifteen samples were selected for Sanger sequencing of the TYRP1b gene, based on PCR amplification success as observed via agarose gel electrophoresis. This assay targets a relatively long DNA fragment (~ 800 bp), making it more sensitive to DNA degradation than shorter PCR assays. Sanger sequencing is a gold-standard method for obtaining high-quality, base-level sequence data, but it requires high-integrity DNA, particularly when amplifying longer fragments. The TYRP1b gene is involved in melanin biosynthesis and has been linked to pigmentation variation in barramundi (*Lates calcarifer*), with potential relevance for phenotypic selection in aquaculture breeding programs (Marcoli et al., 2023). Assessing amplification success and sequencing outcomes from this assay provide additional means for evaluating DNA integrity across extraction methods.

PCR amplification was conducted in 25 µL reactions containing: 2.5 µL of 10× PCR buffer, 0.5 µL of 10 mM dNTP mix (Bioline, London, UK), 1.5 µL of 50 mM MgCl₂, 0.6 µL each of 10 µM forward and reverse primers, 0.1 µL of Taq DNA polymerase (Bioline, London, UK), 18.2 µL of Milli-Q^®^ water, and 1 µL of genomic DNA template.

PCR was performed on a SimpliAmp™ Thermal Cycler (Thermo Fisher Scientific) with the following cycling conditions: initial denaturation at 94 °C for 2 min; 30 cycles of 94 °C for 30 s, 60 °C for 30 s, and 72 °C for 45 s; followed by a final extension at 72 °C for 10 min and an indefinite hold at 4 °C.

Amplification success was first assessed by evaluating PCR product yield and clarity prior to sequencing submission. Samples extracted using the wax-coated dipstick method failed to produce visible bands and were excluded from downstream sequencing. From the remaining methods, three samples showing the strongest amplification were selected from each treatment, yielding a total of 15 samples. These PCR products were submitted to the Australian Genome Research Facility (AGRF) for sequencing.

Across all downstream assays, the same set of 16 individuals was extracted using each method. All 96 resulting DNA samples (16 per treatment) were submitted for SNP genotyping on the Allegro platform. Microsatellite genotyping was performed on all 16 individuals across nine loci. TYRP1b sequencing was performed only on samples that produced a visible PCR product, resulting in differential sample numbers between treatments. These differences reflect assay-dependent amplification success rather than intentional experimental variation.

### SNP Genotyping and Downstream Analyses

A total of 96 samples spanning six DNA extraction treatments were submitted to the Australian Genome Research Facility (AGRF) for genotyping using the Tecan Allegro Targeted Resequencing V2 kit to evaluate their suitability for SNP genotyping in barramundi (*Lates calcarifer*). A 1 µL aliquot of each DNA sample was transferred into a 96-well plate without dilution and processed by the provider. Raw sequencing reads were first trimmed using TrimGalore (v0.4.5; https://github.com/FelixKrueger/TrimGalore) to remove Illumina adapter sequences and low-quality bases (Phred score ≤ 20). Filtered reads were aligned to the *Lates calcarifer* reference genome (v3) using BWA mem (v0.7.17), and probe regions were masked using the samtools ampliconclip function (v1.18). Variant calling was performed within targeted regions between probes using GATK HaplotypeCaller (v4.0.12.0), generating individual GVCF files. These were merged with GATK CombineGVCFs and jointly genotyped using GenotypeGVCFs to produce a single multisample VCF file. Variant depths were extracted using GATK VariantsToTable. Genotypes were recoded from numeric to nucleotide format using PLINK (v1.9.0) with the --alleleACGT and --keep-allele-order flags.

To evaluate the performance of each DNA extraction method, genotype concordance was assessed among replicate samples. Genotype data were processed in R (v4.3.2) using the vcfR and tidyverse packages. Genotype calls were extracted from the VCF file and reformatted to standard categories (homozygous reference, heterozygous, homozygous alternate), with missing data excluded from comparisons. For each individual, pairwise genotype concordance was calculated between the CTAB extraction (used as a reference) and each alternative treatment. Concordance was defined as the percentage of matching genotype calls at non-missing loci. Summary statistics were computed using dplyr, and concordance distributions were visualized using ggplot2.

Statistical analyses were conducted to compare genotype concordance, call rate, and depth among DNA extraction treatments. One-way ANOVAs followed by Tukey’s HSD post hoc tests were used to evaluate differences between treatments. In cases where assumptions of normality or homoscedasticity were not met, Kruskal–Wallis tests and Dunn’s tests were applied using the rstatix and FSA R packages. All plots and statistical outputs were generated in R.

## Results

### DNA Yield

**Fig. 1:**
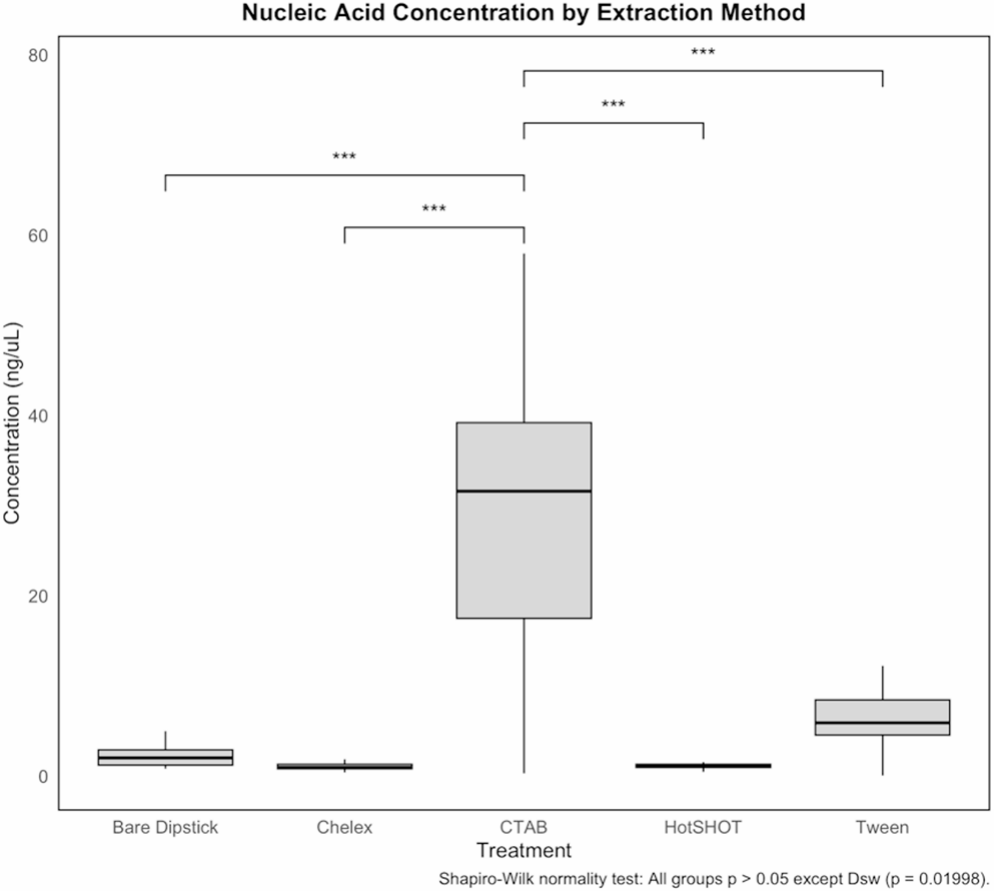
Nucleic acid concentration (ng/µL) by DNA extraction method, measured using the Quantifluor^®^ dsDNA System on a Zephyr^®^ automated workstation. Each sample was measured in triplicate, and mean concentrations were calculated. CTAB yielded significantly higher DNA concentrations than all other methods (mean ± SD: 30.3 ± 17.4 ng/µL), as confirmed by repeated measures ANOVA (F(5, 75) = 40.85, *p* < 2 × 10⁻¹⁶) and post hoc t-tests (***p < 0.001)

The CTAB protocol produced the highest mean concentration of nucleic acids, as measured using the Quantifluor assay, at 30.3 ± 17.4 ng/µL (mean ± SD), substantially exceeding yields from all other methods (Fig. 1; Tables 1 and 2A). In contrast, mean concentrations were 6.5 ± 3.1 ng/µL for the Tween method, 2.2 ± 0.5 ng/µL for the wax-coated dipstick, 1.1 ± 0.3 ng/µL for HotSHOT, 1.1 ± 0.4 ng/µL for Chelex, and 0.5 ± 1.3 ng/µL for the bare dipstick method. A Shapiro–Wilk test indicated that DNA concentrations were normally distributed for all methods except the wax-coated dipstick. To assess differences in yield, a one-way repeated measures analysis of variance (ANOVA) was performed excluding the wax-coated dipstick (F(5, 75) = 40.85, *p* < 2 × 10⁻¹⁶). The effect of the extraction protocol was significant. Post hoc pairwise t-tests revealed that CTAB yielded significantly higher concentrations than Chelex (*p* = 3.7 × 10⁻¹⁵), bare dipstick (*p* = 2.2 × 10⁻¹⁴), HotSHOT (*p* = 3.8 × 10⁻¹⁵), and Tween (*p* = 1.8 × 10⁻¹¹). However, no significant differences were detected among Chelex, bare dipstick, HotSHOT, and Tween (*p* > 0.05 for all comparisons), indicating comparable yields among these methods.

**Table 2:** DNA concentration measured by Quantifluor^®^ fluorescence assay across six DNA extraction methods. Values represent the average and standard deviation (SD) of DNA concentration (ng/µL) based on triplicate measurements per sample. CTAB yielded the highest DNA concentrations, while dipstick-based methods showed substantially lower yields

Extraction Method	Mean DNA Concentration (ng/µL)	Standard Deviation (ng/µL)
Chelex	1.05	0.42
CTAB	30.27	17.47
Bare Dipstick	2.22	1.27
Wax-coated Dipstick	0.52	0.46
HotSHOT	1.07	0.30
Tween	6.52	3.15

### Purity

The CTAB method was the most effective at producing high-purity DNA samples, with 14 of the 16 samples falling within the desired A260/A280 range of 1.8–2.0 (mean = 1.91, SD = 0.26; Table 3). It also yielded the highest A260/A230 ratio (mean = 1.67, SD = 0.46), indicating relatively low levels of organic contaminants and salts. In contrast, the Chelex and HotSHOT methods consistently produced A260/A280 values below 1.8 (means = 1.14 and 1.44, respectively), and low A260/A230 values (0.34 and 0.29), suggesting the presence of contaminants such as proteins or reagents. The bare dipstick and Tween methods showed higher variation in A260/A280 values (SD = 0.89 and 1.00, respectively), with both means falling below 1.8. A260/A230 ratios for these methods were particularly low (bare dipstick mean = 0.08, Tween = 0.70), indicating substantial contamination. Wax-coated dipstick samples were extremely variable in A260/A280 (mean = 7.10, SD = 24.95), likely due to interfering substances. Their A260/A230 ratios were also very low (mean = 0.16, SD = 0.14).

**Table 3:** Mean purity ratios of DNA extracted by six methods, as measured by nanodrop spectrophotometry. The A260/A280 ratio indicates protein contamination (ideal ~ 1.8 for pure DNA), while the A260/A230 ratio reflects contamination from salts or organic compounds (ideal ~ 2.0–2.2). CTAB-extracted samples showed the highest overall purity, while wax-coated dipsticks exhibited extreme variability and poor reproducibility

Protocol	Mean A260/A280	Standard Deviation A260/A80	Mean A260/A230	Standard Deviation A260/A230
Chelex	1.14	0.08	0.34	0.08
CTAB	1.91	0.26	1.67	0.46
Bare Dipstick	1.12	0.89	0.08	0.35
Wax-Coated Dipstick	7.10	24.95	0.16	0.14
HotSHOT	1.44	0.18	0.29	0.24
Tween	1.71	1.00	0.70	1.72

### Integrity

CTAB consistently yielded the most intact, high-molecular-weight DNA as evidenced by Tapestation profiles and DNA Integrity Number (DIN) (Fig. 1A). The number of samples with detectable low molecular weight fragments ranged from 10 (HotSHOT) to 15 (CTAB and wax dipstick). The highest proportion of samples exhibiting a distinct DNA peak above 500 bp was observed for CTAB (15/15), followed by bare dipstick (13/15), and HotSHOT (7/15). In contrast, Chelex, wax dipstick, and Tween demonstrated minimal or no high-molecular-weight peaks, with only 1, 4, and 2 samples exceeding 500 bp, respectively.

**Fig. 2:**
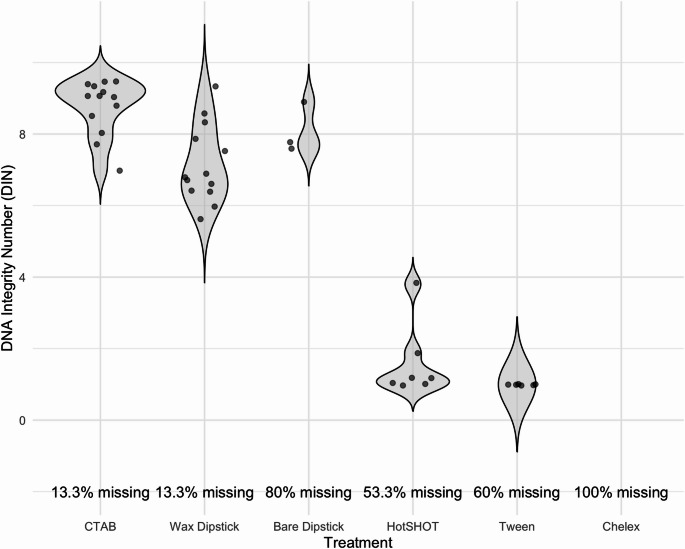
DNA Integrity Number (DIN) distributions for six DNA extraction methods, measured using the Agilent TapeStation with the Genomic DNA ScreenTape assay. DIN values range from 1 (highly degraded) to 10 (highly intact). CTAB consistently yielded the highest-quality DNA (mean DIN = 8.78, SD = 0.77), significantly outperforming all other methods (Kruskal-Wallis χ² = 32.84, df = 4, *p* = 1.29e-06). Chelex, HotSHOT, and Tween produced highly fragmented DNA with mean DIN values near 1, limiting their suitability for downstream genomic analyses. Missing sample percentages reflect the proportion of samples that failed to produce detectable DNA within the assay’s size range, indicating severe degradation or insufficient extraction

The DNA Integrity Number (DIN) values varied significantly across the five DNA extraction methods (Fig. 2). The DIN values ranged from 1 (completely degraded DNA) to 10 (highly intact DNA). CTAB consistently yielded the highest-quality DNA, with a mean DIN of 8.78 (SD = 0.77), reflecting the high-molecular-weight, intact DNA suitable for downstream applications. In contrast, the other methods, including Chelex (mean DIN = 1.00, SD = 0), HotSHOT (mean DIN = 1.59, SD = 1.03), and Tween (mean DIN = 1.00, SD = 0), resulted in significantly degraded DNA, with low DIN values. Dunn’s post-hoc analysis following the Kruskal-Wallis test revealed that CTAB had significantly higher DIN values compared to HotSHOT (p = < 0.0001), Tween (p = < 0.0001), and Chelex (*p* = 0.05). Additionally, the Kruskal-Wallis test showed a statistically significant difference in DIN values across treatments (χ² = 32.84, df = 4, *p* = 1.29e-06). These results indicate that while CTAB extraction consistently produced high-quality, intact DNA, methods like Chelex, HotSHOT, and Tween resulted in degraded DNA, limiting their applicability for downstream genomic analyses.

### Microsatellites

**Fig. 3:**
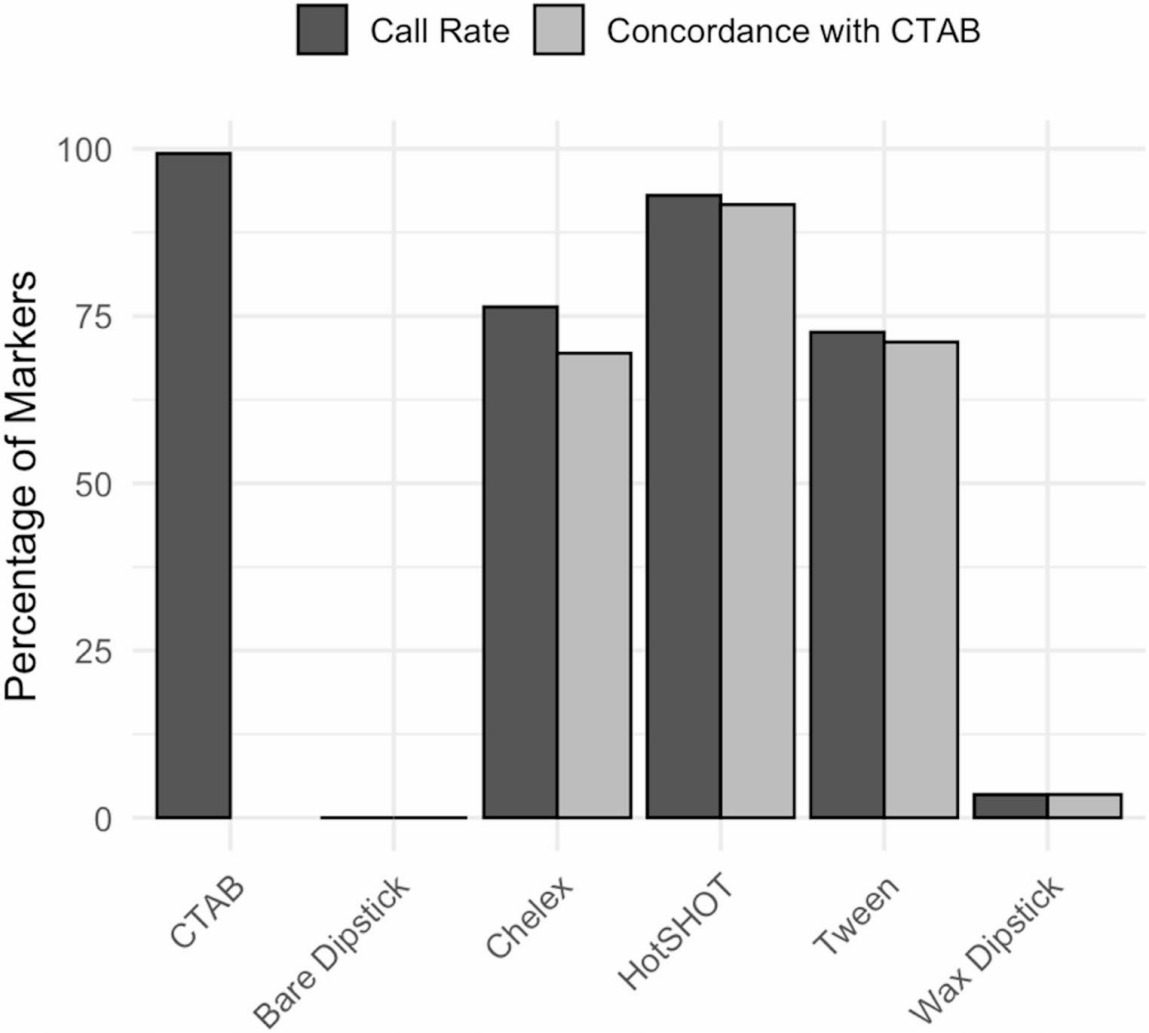
Percentage of nine microsatellite markers successfully genotyped (Call Rate) and concordant with CTAB allele size references across six DNA extraction methods. Call rate represents the proportion of markers producing scorable peaks at expected fragment sizes (150–450 bp), while concordance reflects the percentage of correct allele calls relative to the CTAB benchmark. CTAB achieved the highest call rate (99.3%), while HotSHOT exhibited the highest concordance to the CTAB benchmark. In contrast, both bare and wax-coated dipstick methods had low call rates and minimal concordance, indicating limited suitability for microsatellite genotyping

**Fig. 4:**
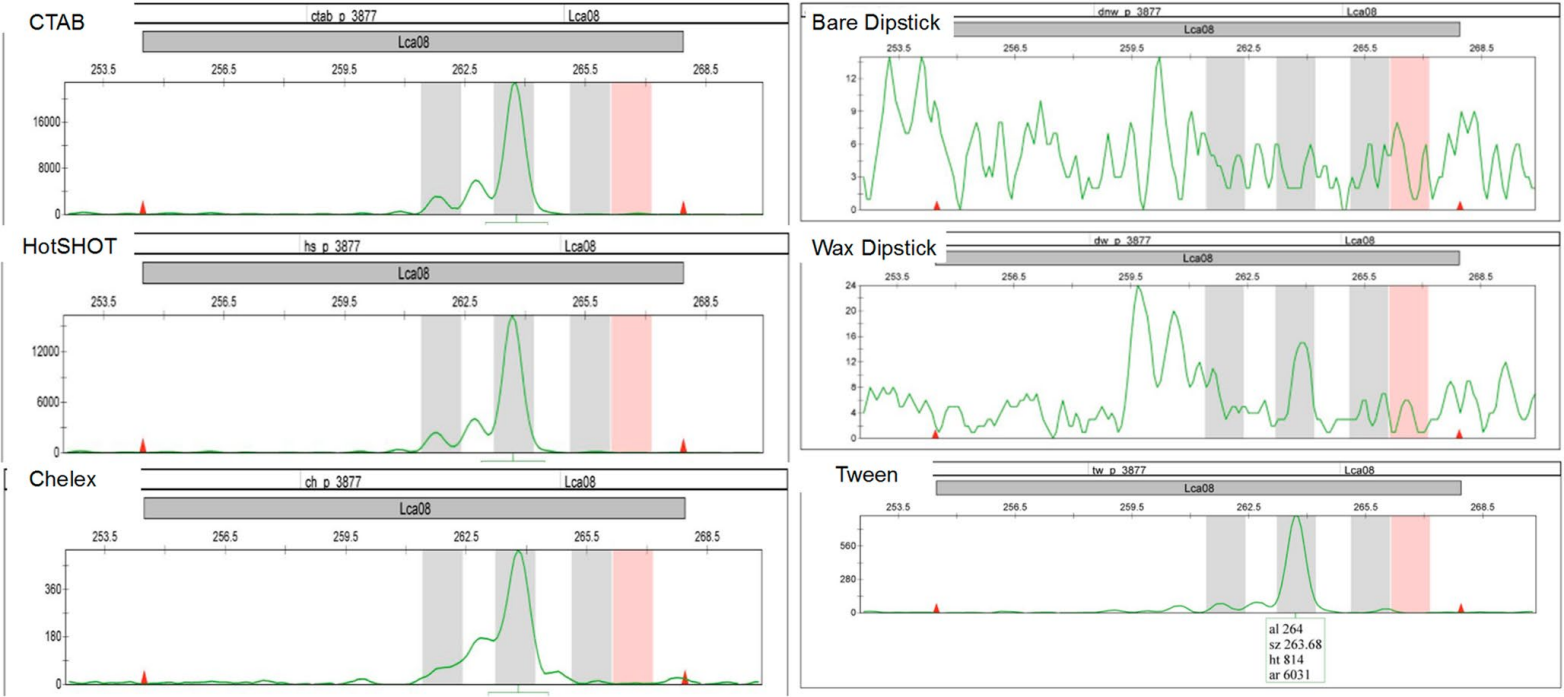
Representative electropherograms from microsatellite genotyping across six DNA extraction methods. The same barramundi fin clip was extracted using CTAB, HotSHOT, Chelex, Tween, wax-coated dipstick, and bare dipstick protocols, then amplified using fluorescently labeled microsatellite primers. Clear, well-defined peaks corresponding to expected allele sizes were observed in samples extracted with CTAB, HotSHOT, Chelex, and Tween. In contrast, electropherograms from the wax-coated and bare dipstick methods displayed erratic, low-amplitude signals with high background noise, indicating poor DNA quality and failed or nonspecific amplification

Microsatellite amplification performance varied considerably among DNA extraction methods (Fig. 3). The CTAB and HotSHOT protocols yielded the most consistent results, successfully amplifying target loci in 99.3% and 93.1% of reactions, respectively. Here, amplification success was defined as the presence of clear, scorable peaks at the expected fragment size, as illustrated in Fig. 4. In contrast, Tween and Chelex extracts showed moderate amplification performance, with overall success rates of 72.6% and 76.4%, respectively, though results varied by locus. For example, amplification success with Tween ranged from 72.6% at Lca58 to 93.1% at Lca20.

To assess amplification accuracy and concordance, allelic size calls from each sample at each microsatellite locus were also compared to those from the corresponding CTAB reference allele genotypes. HotSHOT showed the highest concordance, with 91.7% of allelic calls at loci matching the allele size for its CTAB comparison. Chelex and Tween produced moderate accuracy, with matching calls of 69.4% and 71.1%, respectively. Both dipstick-based methods (bare and wax-coated) performed poorly, with fewer than 4% of loci producing interpretable allele calls that matched CTAB reference allele sizes. These findings highlight CTAB and HotSHOT as the most reliable methods for microsatellite genotyping in this study.

Statistical analysis revealed significant differences in amplification success rates across treatments. The Kruskal-Wallis test for success rate showed a highly significant effect of treatment (χ² = 69.07, *p* = 1.60 × 10⁻¹³), and pairwise comparisons confirmed that CTAB and HotSHOT performed significantly better than other methods, including Chelex and Tween. In contrast, although the Kruskal-Wallis test for correct amplification rates (χ² = 54.13, *p* = 4.96 × 10⁻¹¹) indicated substantial differences across treatments, pairwise comparisons found no significant differences in correct amplification rates between HotSHOT, Chelex, Tween, and wax-coated dipstick treatments (adjusted p-values > 0.48). This suggests that differences in overall performance were primarily driven by amplification success rather than errors in allele size calling once amplification was achieved.

These findings indicate that CTAB and HotSHOT are the most reliable methods for microsatellite genotyping from those approaches evaluated, with CTAB offering the highest amplification success and HotSHOT providing comparable accuracy among successful amplifications. Dipstick-based methods were unsuitable under the tested conditions.

### TYRP1b

Amplification and sequencing success of the TYRP1b gene varied notably across DNA extraction methods (Table 4). Success was defined as generating high-quality bidirectional sequences, specifically those with a high-quality base percentage (%HQ) greater than 85% and strong BLAST alignment to the TYRP1 gene (Expect value ≤ 2.00E-140). The CTAB and HotSHOT protocols consistently yielded the most reliable results, with each producing multiple high-quality sequences that met both criteria. In total, nine samples across all treatments met this quality threshold. The Chelex and bare dipstick extractions each produced two high-quality sequences, although one reverse read from a bare dipstick sample had lower quality (%HQ = 72.6%). In contrast, the Tween method failed to produce any sequences of acceptable quality, with %HQ values near zero. These findings indicate that CTAB and HotSHOT are the most effective extraction methods for successful amplification and sequencing of TYRP1, while Tween was unsuitable for this application. The TYRP1b assay provided an additional test of long-amplicon PCR performance, which remains relevant for trait-associated sequencing and marker validation workflows commonly used in aquaculture breeding programs.

**Table 4:** Sequencing quality metrics for TYRP1b PCR amplicons across DNA extraction methods. Each sample was sequenced in both forward and reverse directions. Reported metrics include the percentage of high-quality bases with a phred score ≥ Q40, post-trim read length, and E-value from BLAST alignment against the expected TYRP1 reference. Values of n/a indicate failed or unusable reads. CTAB and HotSHOT protocols consistently yielded high-quality sequence data, while tween and some dipstick-based extractions frequently failed

Protocol	Sample	Read Direction	% of bases ≥ Q40	Post-Trim Sequence Length	TYRP Expect Value
CTAB	A	Forward	92.70%	674	2.00E-141
		Reverse	94.80%	674	2.00E-166
	B	Forward	91.40%	676	2.00E-141
		Reverse	92.10%	674	1.00E-163
	C	Forward	0.00%	408	n/a
		Reverse	0.30%	321	n/a
Chelex	A	Forward	92.10%	675	5.00E-142
		Reverse	93.80%	674	1.00E-163
	B	Forward	0.00%	426	n/a
		Reverse	0.30%	396	n/a
	C	Forward	92.30%	678	2.00E-141
		Reverse	94.10%	674	1e-163
Bare Dipstick	A	Forward	89.50%	673	2e-140
		Reverse	95.20%	672	2.00E-166
	B	Forward	92.00%	672	2.00E-140
		Reverse	72.60%	332	n/a
	C	Forward	0.00%	443	n/a
		Reverse	n/a	5	n/a
HotSHOT	A	Forward	93.20%	675	2.00E-141
		Reverse	92.40%	674	1.00E-157
	B	Forward	93.60%	674	8e-140
		Reverse	94.50%	673	8.00E-165
	C	Forward	92.60%	675	2.00E-141
		Reverse	93.80%	673	8.00E-165
Tween	A	Forward	0.00%	433	n/a
		Reverse	15.40%	512	n/a
	B	Forward	0.20%	449	n/a
		Reverse	n/a	5	n/a
	C	Forward	0.00%	186	n/a
		Reverse	n/a	5	n/a

### Allegro SNP platform

a) Call rate and read depth

**Fig. 5:**
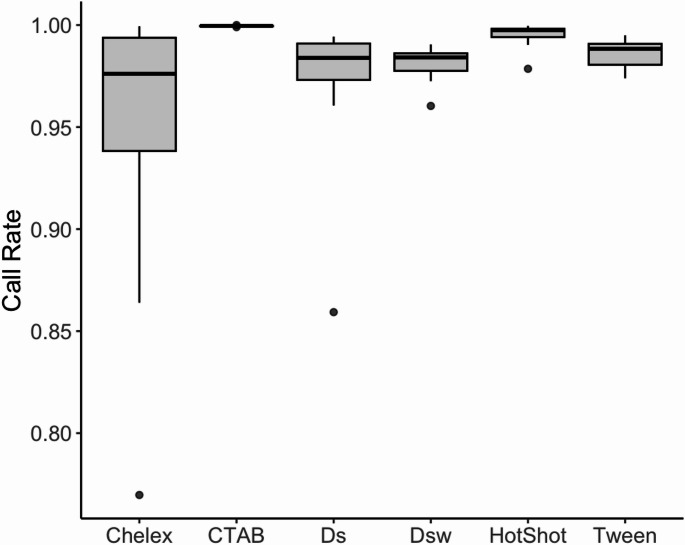
Average SNP call rates for six DNA extraction methods, evaluated using the Tecan Allegro Targeted Resequencing V2 kit. CTAB and HotSHOT produced the highest mean call rates (99.95% ± 0.02% and 99.52% ± 0.52%, respectively). Chelex had the lowest mean call rate (95.23% ± 6.13%) and the highest variability. Statistical analysis (Kruskal-Wallis χ² = 56.041, df = 5, *p* = 7.97e-11) confirmed significant differences among methods, with post-hoc Dunn’s tests indicating Chelex had significantly lower call rates compared to CTAB (*p* = 9.17e-07) and bare dipstick (*p* = 1.00e + 00), while CTAB and HotSHOT did not differ significantly (*p* = 0.4523)

SNP genotyping performance, assessed by average call rate—the proportion of SNP loci with non-missing genotype calls—was highest for the CTAB and HotSHOT methods, reflecting strong reliability and data completeness across replicates (Fig. 5). The CTAB method yielded the highest mean call rate (99.95 ± 0.02%), followed by HotSHOT (99.52 ± 0.52%) and Tween (98.66 ± 0.62%; Fig. 4). The dipstick methods performed moderately well, with call rates of 97.52 ± 3.23% (bare dipstick) and 98.14 ± 0.76% (wax-coated dipstick), respectively. Chelex exhibited the lowest mean call rate (95.23 ± 6.13%) and the highest variability among replicates. Statistical analysis using the Kruskal-Wallis test indicated significant differences in call rates between treatments (χ² = 56.041, df = 5, *p* = 7.971e-11). Post-hoc pairwise comparisons using Dunn’s test with Bonferroni correction revealed significant differences in SNP call rates across several extraction methods. Chelex had significantly lower call rates compared to CTAB (adjusted *p* = 1.92 × 10⁻⁷) and HotSHOT (*p* = 0.006). However, there were no significant differences between Chelex and the dipstick-based methods (bare dipstick, wax-coated dipstick) or Tween, with all adjusted p-values equal to 1.00. CTAB also performed significantly better than the wax-coated dipstick (*p* = 7.80 × 10⁻⁸) and Tween (*p* = 4.82 × 10⁻⁵), but not significantly better than HotSHOT (*p* = 0.452). Bare dipstick outperformed HotSHOT slightly (*p* = 0.017), though the practical difference was small.

Mean sequence read depth per locus varied across extraction methods (Table 5). The bare dipstick method achieved the highest average read depth (284 ± 253 reads per locus), slightly exceeding CTAB (279 ± 293). Chelex (253 ± 370) and Tween (250 ± 178) also showed relatively high read depths. HotSHOT produced a moderately lower mean depth (222 ± 171), and the wax-coated dipstick yielded the lowest mean read depth (190 ± 192). A Shapiro-Wilk test confirmed that read depth data were not normally distributed (W = 0.84647, *p* = 1.66e-08). The Kruskal-Wallis test indicated no significant difference in mean read depth between treatments (χ² = 5.2057, df = 5, *p* = 0.3913), suggesting that all methods provided similar coverage.

**Table 5:** Summary of SNP genotyping performance using the Tecan allegro targeted resequencing V2 platform across six DNA extraction methods. Call rate represents the proportion of SNP loci with successful genotype calls, while read depth refers to the average number of sequencing reads per locus. CTAB and HotSHOT achieved the highest call rates, while the bare dipstick method had the greatest average read depth. Chelex showed the lowest overall performance, with both reduced call rate and high variability in read depth

Protocol	Mean Call Rate	Standard deviation of Call Rate	Mean Read Depth	Standard deviation of Read Depth
Chelex	0.9523	0.0613	253	370
CTAB	0.9995	0.0002	279	293
Bare Dipstick	0.9752	0.0323	284	253
Wax-coated Dipstick	0.9814	0.0076	190	192
HotShot	0.9952	0.0052	222	171
Tween	0.9866	0.0062	250	178

b) Concordance

**Fig. 6:**
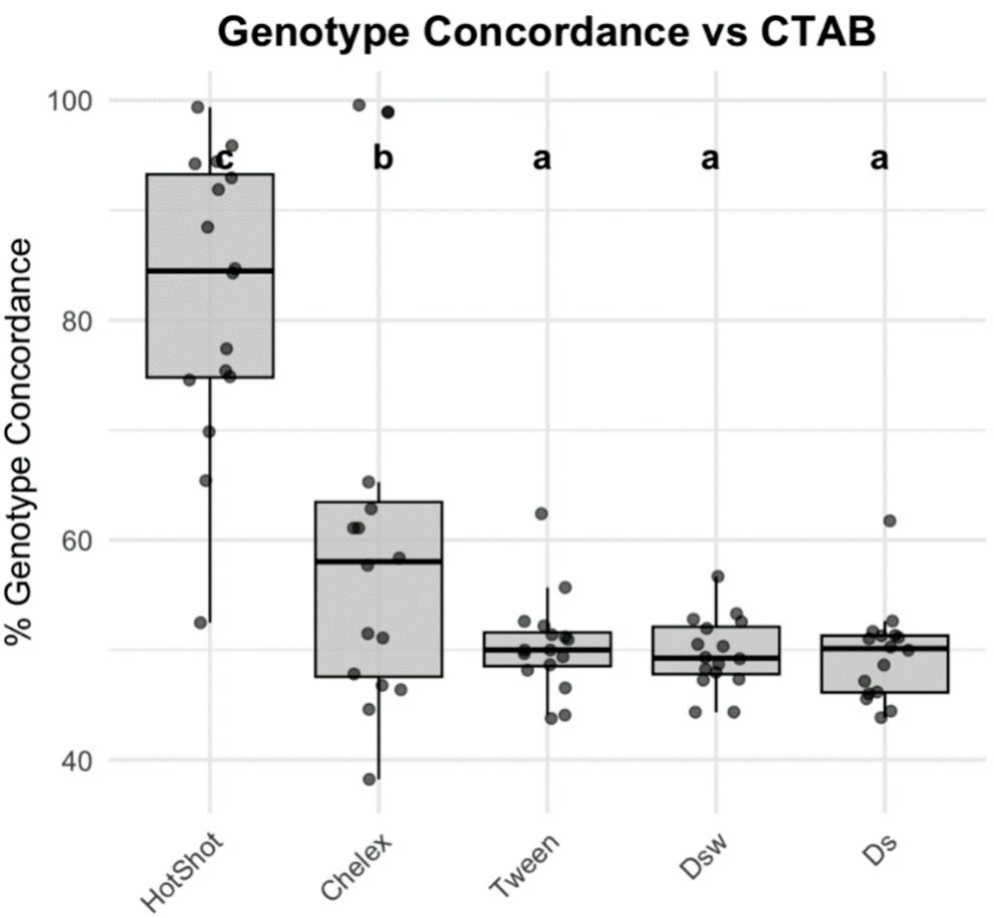
Genotype concordance with CTAB genotype calls for five DNA extraction methods, measured as the percentage of SNP genotypes matching the CTAB reference. HotSHOT achieved the highest mean concordance (82.3%), significantly outperforming all other methods (Linear Mixed-Effects Model, F(4, 60) = 26.03, *p* < 2 × 10⁻¹²). Chelex showed moderate concordance (61.9%), while Tween (50.4%), wax-coated dipstick (49.7%), and bare dipstick (49.6%) methods exhibited significantly lower concordance rates, with no statistical difference among these three treatments. Different letters indicate significant differences between groups (*p* < 0.05, pairwise Wilcoxon signed-rank tests, FDR-adjusted), highlighting HotSHOT as the most accurate alternative to CTAB for genotyping

To evaluate genotyping accuracy across treatments, SNP concordance was measured between replicate samples extracted with each method and those extracted using CTAB, which served as the benchmark due to its known reliability. Concordance with the CTAB reference varied significantly across DNA extraction treatments (Linear Mixed-Effects Model, F(4, 60) = 26.03, *p* < 2 × 10⁻¹²; Fig. 6). HotSHOT yielded the highest mean genotype concordance (82.3%), significantly outperforming all other treatments (*p* < 0.005). Chelex achieved moderate concordance (61.9%), while Tween (50.4%), wax-coated dipstick (49.7%), and bare dipstick (Ds: 49.6%) treatments showed substantially lower and statistically indistinguishable concordance rates. These findings were supported by a non-parametric Friedman test (χ²(4) = 27.85, *p* < 0.0001), and pairwise Wilcoxon signed-rank tests confirmed that HotShot performed significantly better than all other methods (*p* < 0.001, FDR-adjusted). Across all loci and individuals, the HotSHOT method achieved a mean overall SNP accuracy of 82.3% relative to CTAB, while all other rapid methods exhibited substantially lower concordance (50–62%). Collectively, these results indicate that HotSHOT is the most reliable alternative to CTAB for genotyping purposes, while dipstick-based methods performed poorly in this context.

## Discussion

This study compared six DNA extraction protocols—CTAB, HotSHOT, Chelex, Tween, wax-coated dipstick, and bare dipstick—for their suitability in SNP genotyping using the Allegro platform, microsatellite genotyping, and Sanger sequencing of the TYRP1b gene in *L. calcarifer*. CTAB consistently delivered the highest DNA quality and downstream success across all assays but remains time consuming and resource intensive. HotSHOT, despite yielding highly degraded DNA, performed remarkably well in all genotyping applications, confirming the Allegro platform’s tolerance to low-integrity DNA and establishing HotSHOT as a practical alternative for high-throughput use. In contrast, Chelex, Tween, and both dipstick-based methods showed poor reproducibility and weak downstream performance, limiting their current utility for large-scale aquaculture genotyping.

### Extraction Method Performance

CTAB consistently yielded the highest-quality DNA across all metrics, including concentration (~ 30 ng/µL), purity (A260/A280 ~ 1.9), and integrity (DIN ~ 8.8). These values reflect effective removal of proteins, polysaccharides, and other inhibitors commonly found in mucous-rich tissues such as barramundi fin clips (Murray and Thompson 1980; Poompuang and Hallerman 1997). The resulting DNA quality meets or exceeds the recommended input standards for the Allegro platform, and accordingly, CTAB-extracted samples achieved near-complete SNP call rates (> 99.9%). While CTAB provides an ideal benchmark, understanding the Allegro platform’s tolerance for lower-quality DNA requires comparison with results from the other extraction methods.

An important limitation of this study is that DNA input amounts were not normalized across extraction methods. Each protocol was applied in its conventional form—including its typical tissue input and final elution volume—to reflect how these methods would realistically be used in hatchery or high-throughput genotyping contexts. As a result, CTAB extractions originated from ~ 100 mg fin tissue and yielded concentrated DNA, whereas rapid methods used ~ 1 mg tissue and differed substantially in final extraction volume (e.g., 140 µL for HotSHOT vs. 500 µL for Chelex). These differences inevitably influenced downstream performance and partly explain the comparatively poor results from Chelex and Tween. Concentrating low-yield extracts or normalizing DNA inputs for all assays would likely improve genotyping accuracy for some methods; however, such modifications were beyond the scope of this study and would reduce the relevance of the results for real-world high-throughput applications.

The HotSHOT method produced highly degraded DNA (DIN ~ 1.6) with low yield (~ 1.1 ng/µL), yet still performed strongly in genotyping applications. HotSHOT achieved high call rates (99.52%), strong microsatellite amplification (93.1%), and the highest genotype concordance with CTAB references, indicating strong accuracy even with degraded DNA. This suggests that the Allegro platform is relatively tolerant of fragmented DNA, likely due to its reliance on short probe extension and ligation. However, low-molecular-weight DNA may reduce library preparation efficiency and increase the risk of dropout or allele imbalance (Head et al. 2014). Such imbalance may lead to biased SNP calls, including underrepresentation of one allele in heterozygous loci (so called null alleles), potentially compromising genotype accuracy in applications like parentage assignment or genomic selection (Head et al. 2014). As such, the HotSHOT method should be validated against a more reliable method before large-scale implementation. These findings support HotSHOT as a viable low-cost, high-throughput method when time and budget are constraints, even though its utility is limited for long-read sequencing or applications requiring high molecular weight DNA.

In contrast to CTAB and HotSHOT, the Chelex, Tween, and dipstick-based protocols were unsuitable for Allegro SNP genotyping due to low DNA integrity, inconsistent purity, and poor downstream performance. Chelex-extracted DNA exhibited low A260/A280 (~ 1.14) and A260/A230 ratios, indicative of salt or protein contamination, consistent with prior studies showing that residual Chelex resin can interfere with enzymatic reactions (Walsh et al. 1991; Singer-Sam et al., 1989). Although Chelex occasionally enabled genotyping, its variability in quality and purity made it unreliable for high-throughput or critical applications. Tween-extracted DNA showed slightly higher yields than Chelex or HotSHOT but suffered from similarly degraded integrity (DIN < 2) and variable purity, likely due to detergent carryover. Both wax-coated and bare dipstick methods performed poorly overall, yielding very low DNA concentrations (< 2 ng/µL), high variability in purity (A260/A280 SD = 24.95 for wax-coated), and minimal success in PCR and SNP assays. While DIN values were occasionally high when yields surpassed detection thresholds, this did not translate into usable sequencing libraries. Notably, the dipstick protocols published by Zou et al. (2018) and Mason and Botella (2020) primarily introduced the physical dipstick design; lysis and binding buffers were not standardized and often left to users. The lysis buffer used in this study was based on suggestions from these authors, but it may not have been optimal for fin tissue from barramundi. The poor performance observed here may reflect incompatibility between lysis chemistry and fish mucus or connective tissue, rather than the dipstick format itself. Further optimization—particularly of lysis conditions and potential cleanup steps—may improve dipstick performance for animal tissue genotyping. Nonetheless, in their current form, these protocols were not compatible with Allegro, which requires DNA free from inhibitors to ensure efficient probe hybridization and ligation.

### HotSHOT for Aquaculture Applications

HotSHOT’s utility has been demonstrated across diverse taxa and sample types, including mouse tissue (Truett et al. 2000), invertebrate eggs (Montero-Pau et al. 2008), processed fish products (Labrador et al., 2019), and insect trap samples (Fowler et al. 2023). In these studies, the common denominator was PCR-based downstream analysis—highlighting HotSHOT’s compatibility with workflows that do not require long DNA fragments, but instead rely on clean amplification of short targets. This aligns with our findings that HotSHOT is suitable for the Allegro platform and PCR-based microsatellite genotyping and TYRP1 gene sequencing.

From a practical standpoint, the trade-offs between DNA quality, processing time, and cost are critical in high-throughput breeding programs. CTAB, while producing the highest-quality DNA, was also the most resource- and labor-intensive method evaluated. Material costs alone range from AUD $5 to $6 per sample, amounting to $5,000–6,000 for 1,000 samples, excluding labor. The protocol involves hazardous reagents, extensive centrifugation, and multiple solvent extraction steps requiring fume hood access. When performed manually by a single technician in 24–48 sample batches, processing 1,000 samples may take 10–15 working days, or 2–3 weeks of full-time effort. Factoring in labor (e.g., technician time, waste disposal, infrastructure overhead), the total cost could rise by an additional $2,000–3,000, making CTAB a costly option for large-scale applications. Despite these limitations, CTAB remains widely used in molecular ecology due to its reliability across diverse tissue types (Doyle and Doyle 1987; Schiebelhut et al. 2017).

In contrast, HotSHOT extraction represents a highly cost-effective and scalable alternative. With a material cost of approximately $0.30 AUD per sample, the total reagent and consumable cost for 1,000 samples is less than $300 AUD. The protocol is fast (under 2 h), does not require centrifugation or hazardous chemicals, and is compatible with automation in 96-well plate formats. Originally developed for mouse genotyping, HotSHOT has since been adopted in high-throughput pipelines due to its simplicity and reliability (Truett et al. 2000). A single technician can process 200–400 samples per day, allowing 1,000 samples to be completed in 3–5 days. This substantial time and cost saving makes HotSHOT particularly attractive for routine genetic screening in hatchery operations.

Similarly, Tween-based extractions, when conducted in tubes rather than plates, also cost approximately $0.30 per sample, though the total processing time is slightly longer (4–5 h). Chelex was moderately priced at ~$1.50 per sample, with low hands-on time but limited compatibility with automation. The bare and wax-coated dipstick methods also offer inexpensive workflows ($0.60–0.65 per sample) using minimal reagents and standard lab plasticware. However, these protocols required overnight incubations and more bench handling than HotSHOT or Tween, limiting their throughput despite their low material cost. Dipstick extraction methods have been highlighted as promising for field-based DNA extraction with minimal equipment (Zou et al., 2018), but may be less practical for large-scale breeding programs.

Overall, the findings suggest that HotSHOT strikes the best balance between cost, scalability, and turnaround time. It is well suited to large-scale applications such as selective breeding programs, where thousands of DNA extractions may be required per generation. The ability to reduce reagent costs by over 90% compared to CTAB, while also saving substantial technician time, enables more frequent and accessible genetic monitoring in aquaculture settings. As high-throughput genotyping continues to expand in aquaculture (Houston et al. 2020; Robledo et al. 2018), the need for cost-efficient and automation-compatible extraction protocols will become increasingly important.

While CTAB remains the method of choice when DNA quality is paramount—for example, in genome assembly or long-read sequencing—HotSHOT provides a compelling second-choice option for routine, large-scale genotyping in aquaculture. Its affordability, speed, and proven compatibility with PCR and targeted sequencing applications make it an attractive solution for genetic monitoring, parentage verification, and genomic selection in breeding programs.

### Practical Recommendations and Future Directions

This study highlights the importance of evaluating DNA extraction protocols within the specific context of aquaculture genotyping workflows. While the CTAB method remains the gold standard for DNA quality, consistently delivering high yield, purity, and integrity, it is not well suited for large-scale or routine use due to its time-consuming protocol, reliance on hazardous reagents, and higher cost. In contrast, HotSHOT emerges as a practical second-choice method: it is inexpensive, fast, and easy to implement, and it performed reliably across all tested downstream applications, including SNP genotyping, microsatellite amplification, and gene sequencing.

Methods such as Chelex, Tween, and dipsticks showed lower consistency and DNA quality and are not currently recommended for high-throughput genotyping without further modification. However, they remain of interest for field-based or rapid diagnostic workflows due to their simplicity and low cost. Future work should explore the potential to improve these rapid methods through the inclusion of cleanup steps (e.g., magnetic bead purification) or buffer optimization to enhance purity and reproducibility. Additionally, simplified or automated CTAB variants could offer a middle ground for balancing DNA quality and throughput.

Several alternative DNA extraction methods show promise for large-scale SNP genotyping using platforms like Allegro, particularly in aquaculture species such as barramundi where high sample throughput, cost-efficiency, and minimal infrastructure are essential. Commercial kits such as Q-Extract (Ampliqon, 2023) and Extracta (Quantabio, 2021) offer rapid, two-step protocols that produce PCR-ready DNA in under 30 min without the need for hazardous chemicals, making them scalable and suitable for automation. Similarly, simple boiling protocols have been successfully applied to fish tissues for conventional and real-time PCR, offering an extremely low-cost solution with acceptable DNA quality for short-read assays (Xiong et al., 2019). FTA^®^ cards (Qiagen, 2022) provide a field-friendly option for sample preservation and transport, enabling long-term, room-temperature DNA storage and recovery suitable for PCR-based genotyping. While these methods may produce fragmented DNA or lower yields, they may be compatible with short-fragment applications like Allegro. Future validation of these methods in fin tissue from aquaculture species would help refine DNA workflows tailored to genomic selection, parentage assignment, and large-scale genotyping in breeding programs.

A summary of overall method performance across yield, purity, integrity, and downstream applications is provided in Table 6. In conclusion, HotSHOT offers a cost-effective and scalable solution for high-throughput SNP genotyping in barramundi and similar species, particularly when paired with robust platforms like Allegro that tolerate moderate DNA fragmentation. While CTAB continues to be the method of choice for maximum data fidelity, the results of this study clarify the trade-offs between DNA quality, processing time, and cost. These insights can guide the design of efficient, evidence-based breeding pipelines in both commercial and research settings.

**Table 6:** Each method was evaluated based on DNA yield, purity (A260/A280 and A260/A230), integrity (DIN), SNP genotyping performance (call rate and concordance with CTAB), and success in microsatellite and TYRP1 gene sequencing assays. Symbols represent relative performance: +++ (high), ++ (moderate), + (low), – (poor), and n/a (not applicable or not tested)

Method	Yield	Purity	Integrity	SNP Call Rate	Concordance with CTAB	Microsatellite	TYRP1 Seq
CTAB	+++	+++	+++	+++	n/a	+++	++
HotSHOT	+	++	+	+++	+++	++	+++
Chelex	+	+	–	+	++	+	++
Tween	+	–	–	++	+	++	–
Dipstick (wax)	+	–	++	++	+	–	n/a
Dipstick (bare)	+	–	+	++	+	–	+

### Limitations

This study has several limitations that should be considered when interpreting the results. First, tissue inputs and extraction volumes differed substantially between protocols, reflecting their typical usage but preventing direct normalization of DNA amounts across methods. These differences likely contributed to variable downstream performance, particularly for low-yield methods such as Chelex and Tween. Second, DNA inputs were not concentrated or cleaned, and no post-extraction normalization was performed; modifying these protocols could improve genotyping outcomes but would reduce their suitability for rapid, high-throughput workflows. Third, the study was constrained to a sample size of 16 individuals and did not include commercial rapid-extraction kits or magnetic-bead methods, which may offer intermediate performance. Fourth, TYRP1b sequencing was only attempted on samples that successfully amplified, resulting in unequal sample sizes between treatments. Finally, although fin clips had been stored for approximately 12 months, only CTAB extracts were confirmed to have high-molecular-weight DNA; thus, subtle degradation effects in rapid methods cannot be entirely excluded. These limitations underscore the need for future work that incorporates standardized DNA inputs and evaluates additional extraction chemistries.

## Data Availability

The data that support the findings of this study are available from the corresponding author upon reasonable request. Due to third-party restrictions on commercial sample use, some raw genotyping data cannot be publicly shared.
